# Breaking from the past? Environmental narratives, logics of power, and the (re)production of food insecurity in South Sudan

**DOI:** 10.1111/disa.12658

**Published:** 2024-10-23

**Authors:** Francois Sennesael, Harry Verhoeven

**Affiliations:** ^1^ University of Oxford United Kingdom; ^2^ Columbia University United States

**Keywords:** Sudan, environmental narratives, famine, food security, South Sudan, water–energy–food (WEF) nexus

## Abstract

Skyrocketing commodity prices and conflict‐induced mass hunger in recent years have resuscitated discussions about why famines frequently reoccur in specific spaces of vulnerability. Intervention efforts still too often isolate food (in)security from its interwovenness in the political economy of water and energy and from the role of ideas in forging these interconnections across long time periods. Using (South) Sudanese history to rethink the causes of recurrent food insecurity, we underscore the need to analyse how political elites imagine the role of the water–energy–food nexus and associated environmental narratives in consolidating power. South Sudan's 2011 secession (from Sudan) marked the culmination of a struggle against a state that insurgents regarded as having starved its citizens. However, since independence, its leaders have replicated the nostrum they once combatted: Sudanese resources must ‘feed the world’. A fixation with inserting water, energy, and food resources into global markets infuses their strategy, even if such an approach will not engender food abundance.

## 1 INTRODUCTION

In 2023, acute food insecurity impacted more than 281 million individuals across 59 countries, with 705,000 of them experiencing catastrophe‐level conditions (Food Security Information Network, 2024). The articulation of myriad famine prevention frameworks and the establishment of early warning systems notwithstanding, severe food insecurity endures, especially across Africa where ‘[i]n 2022, 19.7 percent of the population was undernourished, an increase of 4.6 percentage points since 2010’ (FAO et al., 2023, p. 2). The persistence of both recurrent and acute food insecurity predated the COVID‐19^1^ pandemic, which exacerbated the vulnerability of an array of social groups but did not cause the permanency of severe hunger for hundreds of millions of people (Clapp and Moseley, 2020). Despite (or perhaps because of) the political salience of the problem, food insecurity still tends to be widely framed as a detached technical issue, necessitating technocratic solutions that deliberately steer clear of politics (Moragues‐Faus and Marsden, 2017). The upshot is all too frequently that these so‐called scientific approaches are embedded in enduring stereotypes and poorly evidenced, ahistorical causal logics that fail to acknowledge how the struggles of people to access food—and their connections with water and energy systems—have been integral to the projection of power by local elites in states around the world (Watts, 2013; Chadwick, 2019). They also overlook how the act of defining hunger—and therefore also determining how (not) to respond to it—contributes to what Sell and Williams (2020) term ‘a global political economy of structural pathogenesis’ in which the authority and wealth of some are predicated on the malnutrition of many others.

In this paper, we borrow from critical scholarship that draws attention to the social processes that render populations vulnerable to starvation. Findings from mixed‐methods research emphasise the importance of social organisation in resisting predatory state institutions and preventing deadly hunger (Maxwell et al., 2016; Cao, Xu, and Zhang, 2022); by contrast, administrative decisions and humanitarian initiatives that inhibit local adaptation strategies can dramatically heighten the susceptibility of the undernourished to infections (de Waal, 1989). However, *how* deadly hunger is mismanaged also begs the equally critical question of *why* it tends to reoccur in specific states and societies. The reality of severe hunger as a recurrent, collective condition suggests that food crises should be thought of not only in relation to victims, but also beneficiaries who recurrently take advantage of the shifting terms of exchange ‘when the stigmata of starvation become visual’, as Rangasami (1985, p. 1747) observed.

We argue here that the role of ideas about the interplay between food policy, development, and state‐building is critical in reproducing food insecurity in the Horn of Africa and, indeed, elsewhere. Moreover, we propose that both acute and chronic crises are better understood by studying their embeddedness in the historically contingent interlinkages between water, energy, and food systems and the imaginaries that have moulded—and continue to mould—how these systems function. The question of why hunger tends to be so recurrent in specific polities demands a reckoning with the political interests that thrive off the precarity of the rural poor and the displaced and whose jostling for power can occasionally facilitate food relief but also sometimes plunge populations into famine (Bush, 1996; Deng, 2021; Jaspars, Majid, and Adan, 2023). As we demonstrate, these interests are not just a function of material circuits of accumulation, crucial as these are. We also emphasise the interlinkages between the consolidation of political authority and so‐called environmental narratives—evocative storylines disseminated by bureaucratic and epistemological elites that legitimise far‐reaching decisions on how water, energy, and food resources are used and distributed. These help to explain how state institutions actively skew the playing field and prioritise highly exclusionary operationalisations of water, energy, and food security that, despite their manifest inefficiencies and regressive effects, endure in high‐level frameworks and discourses, national policies, and quotidian administrative decisions.

To be sure, international organisations have, to some extent, acknowledged the nefarious effects of dysfunctional institutions and their role in perpetuating hunger, as evident in the proliferation of capacity‐building programmes aimed at ‘fixing’ state fragility and of applied political economy analysis in food policy planning (Swinnen, 2010). Global discourses on food security have become enmeshed within a humanitarian–development–peace nexus approach that foregrounds hunger both as a major driver of political instability and a consequence of conflict (World Food Program USA, 2017). They have increasingly recognised the entanglements between water resources, the energy system, and food production (the ‘water–energy–food (WEF) nexus’) and the need for systemic policies that target these connections in an era of worsening climate change (Abbott et al., 2017; Hasegawa et al., 2021). Yet, such initiatives notwithstanding, interventions in the world's most food‐insecure locations continue only to incorporate tepidly the decisive role of local elites as drivers of food insecurity and the role that food—and its interlinkages with water and energy—plays in cementing their hold on economic and political power, thereby diminishing the effectiveness of prevention efforts (see, for example, World Bank Group, 2015).

This paper centres on one of the world's most perilous ‘spaces of vulnerability’, (South) Sudan, to rethink the ways in which exclusion and hunger to the point of starvation continue to occur, beyond what one colonial mandarin called the ‘annual lottery of the harvest’ (Watts and Bohle, 1993, p. 64). Material choices and structures are fundamental to how access to food is persistently unsettled in many spaces of vulnerability. But food shortages have not only presented rent‐seeking opportunities for traders and state officials or served as a disciplinary tool against rebellious populations. Great hungers have also been driven by the Pax Britannica's economic liberalism and its Malthusian and Smithian dogmas—such as in India during the *Late Victorian Holocausts* of the nineteenth century (Davis, 2001)—and have underpinned the erection of new ideological utopias and political structures. Joseph Stalin's Soviet Union and Democratic Kampuchea are cases in point (Tyner and Rice, 2016). That said, ideas can also be majorly impactful in ways that are less ostensibly doctrinaire or less obviously couched in world historical ‘isms’. This paper highlights the longitudinal role of hegemonic ‘environmental narratives’ that link persistent ideas about the management of water, energy, and food resources, and the consolidation of political authority. Environmental narratives are widely‐held, simplified accounts of environmental change: while the evidence for the causal connections they identify is frequently febrile, these ideas persist over time and are presented as scientific—and therefore authoritative and apolitical. In the Horn of Africa and elsewhere, environmental narratives have buttressed a range of policies that constrain behaviour and forms of social organisation asserted to be detrimental to ecological sustainability while privileging interventions regarded as essential to a very specific interpretation of development (Verhoeven, 2011). In that vein, this paper concentrates on the historical antecedents and formative socialisation that undergird how elites ‘see’ and exploit interlinkages between water, energy, and food as a political instrument in power struggles and the attempted extension of state authority.

To highlight how the politics of environmental narratives underpin structural food insecurity, we anatomise the case of South Sudan. Its history is marred by severe and persistent lack of access to food among a range of societal groups. From 1983, the rebels of the Sudan People's Liberation Movement/Army (SPLM/A) championed a socialist, democratic revolution, advocating for ‘bringing towns to the people’ as liberation praxis to eliminate omnipresent hunger. This slogan strategically countered the authoritarian approach of successive Sudanese regimes to development through extractive and centralising control over water, energy, and food, which entrenched extreme poverty.

Thirteen years after the SPLM/A led the people of South Sudan to independence (gained on 9 July 2011) and six years after a devastating civil war, the ruling party claims to be articulating a new political–economic strategy. We argue that this plan, strikingly, prioritises the same economic recipes and high‐modernist outlook regarding export agriculture and dam building that the liberation movement historically denounced. Moreover, while the SPLM/A promises a ‘green revolution’ that will simultaneously address food insecurity and energy poverty, more than 63 per cent of South Sudan's population finds itself in IPC Phase 3 or worse (FSIN, 2023, p. 134).^2^ Leveraging extensive fieldwork in both Sudan and South Sudan, this paper illustrates why a fixation on inserting water, energy, and food resources into regional and global markets has become a key plank of the SPLM/A's post‐conflict strategy, even if doubling down on the same approach and expecting different results will not engender the food abundance promised to citizens and international partners. We contend that what explains this re‐embrace of a timeworn environmental narrative is the socialisation of South Sudan's elites into seeing extraversion around water, energy, and food as a ‘natural’ approach to state‐building, combined with the proven utility of such a stratagem as an instrument for capturing rents and controlling political power.

The paper begins by introducing the literature on the interconnections between water, energy, and food systems and its usefulness in rethinking food insecurity, especially if thinking in terms of a WEF nexus is supplemented with historicising and politicising how those linkages have been forged. We concretise this approach by outlining the function of environmental narratives in state‐building in Sudan and the centrality of widespread hunger as both a driver and consequence of the concentration of political and economic power. Subsequently, we explore the SPLM/A's aspirations today, examining how oil, hydropower, and agriculture are woven into its post‐conflict articulation and management of sovereignty in South Sudan. Lastly, we reflect on how this analysis can help to address the structural drivers of food insecurity across the Horn of Africa.

## 2 FOOD (IN)SECURITY, NEXUS IMAGINARIES, AND ENVIRONMENTAL NARRATIVES: CONCEPTUAL TERMS OF ENGAGEMENT

This section assesses how thinking about food insecurity evolved from a problem studied in relative isolation to one increasingly approached, in the wake of turbulence in global commodity markets in the early twenty‐first century, as part of a WEF nexus. We recognise the merits of this conceptual shift towards integration while flagging the need to historicise and politicise how and why particular interconnections have been constructed rather than considering them to be obvious. Such a recasting of how water, energy, and food systems interact in socially‐constructed ways dovetails with the work of scholars who critically probe orthodoxies in the stewardship of natural resources and pinpoint how accompanying simplified accounts of environmental change mask undemocratic practices and their distributional consequences.

From the modernisation paradigm emphasising economic growth and agricultural productivity in the 1950s and 1960s to human‐rights based approaches in the 1990s, myriad frameworks have emerged in the pursuit of understanding food insecurity and its remedies (Sassi, 2018). The 2005–08 global crisis, during which the prices of food, energy, and land simultaneously spiked, marked a critical juncture. Until then, food (in)security had been addressed mostly in isolation; despite incipient approaches within international organisations that recognised linkages with water and energy, policy fragmentation and siloisation remained predominant (Benton, 2016). However, the food crisis of the 2000s accentuated the vulnerability of emerging economies to the volatility of global commodity markets and the ways in which concurrently rising demand for oil, gas, and biofuels, as well as land, simultaneously hit developing countries on the production side (making it more expensive to produce food) and the consumption side (curtailing access to food for hundreds of millions of people as more liberalised and globalised markets allowed price hikes to be rapidly transmitted) (McMichael, 2009). Moreover, these anxieties were reinforced by concerns over disruptions of the water cycle in Africa, Asia, and the Middle East that risked sapping the potential of producers, whose capacity to adapt to climatological volatility had already been battered by years of neoliberal structural adjustment programmes. The renewed interest spurred by the crisis thus increasingly translated into ‘food systems’ approaches pioneered by international institutions that placed food security in a wider framework (Westengen and Banik, 2016).

The framing of water, energy, and food as fundamentally intertwined policy areas was rapidly mainstreamed, especially through the ‘World Economic Forum Annual Meeting 2008’ in Davos, Switzerland, and ‘The Water, Energy and Food Security Nexus – Solutions for the Green Economy’ conference in Bonn, Germany, in 2011. The notion of hard‐wired linkages between water, energy, and food, which can be measured and planned for by scholars, investors, and administrators, appealed to many different constituencies (Bazilian et al., 2011). Thus understood, ‘the’ nexus offers policymakers the prospect of actionable trade‐offs between output and consumption in an age of climate change (Rasul and Sharma, 2016). Thinking in terms of these interlinkages is vital for global planning: with demand for food production expected to increase significantly, policymakers need to prepare for simultaneously escalating demand for water, because nexus analyses underline that agriculture accounts for 70 per cent of total freshwater withdrawals worldwide. Concurrently, as a landmark report of the Food and Agriculture Organization of the United Nations (FAO) noted, ‘the food production and supply chain consumes about 30 percent of total energy consumed globally’ (FAO, 2014, p. 1)—underlining the importance of not only water and food systems in mitigating greenhouse gas emissions and stabilising energy prices, but also the momentousness of energy in rendering agriculture and water resources management more affordable and sustainable.

This approach resonated because it coincided with debates at the United Nations over the post‐2015 development agenda and surging demand for resources owing to decades of intensifying globalisation. Its advocates saw the WEF nexus as crucial to the Sustainable Development Goals, providing science‐based solutions to global hunger, risks of resource wars, and sluggish economic growth (Cairns and Krzywoszynska, 2016). *Integrated*, *systemic*, and *holistic* approaches became indispensable concepts in the programming of food security projects within non‐governmental and international organisations, as the WEF nexus infused a new wave of scientism into debates (Wiegleb and Bruns, 2018; Müller et al., 2020). Policymakers and scholars working within this paradigm have predominantly embraced a techno‐scientific, positivist, and ‘solution‐oriented’ approach. They champion the data‐driven integration of markets to address scarcity and hunger—as scaling and fostering interdependence are argued to systemically hedge against shocks and to lower economic and environmental risks for producers and consumers alike.

While nexus scholarship should be credited with impelling more interdisciplinary approaches to food (in)security and encouraging a reckoning with how water and energy policies influence the prevalence of hunger (Allan, Keulertz, and Woertz, 2015), the notion that science and economics can ‘solve’ the inequities that arise from the entanglements of the water, energy, and food sectors, is fanciful. ‘The’ WEF nexus is not a deterministic web of connections that exists in a politically‐agnostic universe and which policymakers can readily rationalise to tackle food insecurity. Critics have challenged the blindness of nexus framings of how the poor experience and perceive of resource scarcity and the failure of much of the nexus literature to incorporate adequately sustainable livelihoods for economically vulnerable and politically marginalised populations into policy pathways (Biggs et al., 2015; Simpson and Jewitt, 2019). WEF nexus accounts routinely overlook the importance of historical path dependencies in moulding how institutions shape not only interactions between water, energy, and food systems, but also what types of integration they prioritise and consider as effective. In some parts of the world, such as the Nile Basin, political elites have long imagined various nexuses (plural) between water, food, and (to a lesser extent) energy, and have sought to naturalise and institutionalise the complementarities and trade‐offs between them (Verhoeven, 2015a). The crafting (or fading) of any nexus needs to be situated within specific cognitive, social, and political environments; doing so can help to explain its persistence over time, as it underlines the role of ideas and conceptions of power that lay behind it.

Historicising and politicising not only the material intersections between water, energy, and food, but also the way in which specific connections are talked about and presented as natural and self‐evident, aligns closely with the scholarship on environmental narratives. The latter has endeavoured to deconstruct powerful orthodoxies that offer simplified explanations of environmental change and that blame shortsighted developmental practices—usually by illiterate rural dwellers—for vicious cycles of ecosystem degradation and underdevelopment (Nygren, 2000). In the classic formulation of Leach and Mearns (1996, p. 1): ‘The driving force behind much environmental policy in Africa is a set of powerful, widely perceived images of environmental change. They include overgrazing and the “desertification” of drylands, the widespread existence of a “woodfuel crisis”, the rapid and recent removal of once‐pristine forests, soil erosion, and the mining of natural resources caused by rapidly growing populations. So self‐evident do these phenomena appear that their prevalence is generally regarded as common knowledge among development professionals in African governments, international donor agencies, and non‐governmental organizations’.

The dominant status of such narratives about the causes and effects of development and environmental change, then, has less to do with the actual empirical evidence buttressing them, which is routinely absent or points in a different direction (see, for example, Adams et al., 2004; Lane, 2009; Svarstad and Benjaminsen, 2017), and more with the crystallisation of received wisdom around key assumptions and explanatory logics that aid the (re)production of certain social orders (Roe, 1991). A principal effect of such discourses tends to be legitimating the reservation of decision‐making power in the hands of a select group of savants, at the expense of local communities deemed to lack requisite knowledge. Thus understood, the concept of environmental narratives opens new perspectives on why policies with disastrous results nonetheless persist and facilitates the disassembling of how administrative, economic, and political elites entrench their authority via discursive strategies.

Crucial to this process is the obfuscation of how unequal access to natural resources has been historically established. For instance, in North and East Africa, European colonialism was accompanied by environmental narratives of deforestation, desertification, and overgrazing, which emphasised the colonial state's duty to alter profoundly the food production and water management practices of indigenous populations in the name of rationally managing fragile environments (Anderson, 2002; Davis, 2007). Similarly, the narrative of water scarcity became vital to strengthening the state of Israel after 1948 and solidifying a still inchoate nation, not only because it helped veil the displacement and disenfranchisement of Palestinian inhabitants of the territory now under Israeli control, but also because of how it justified the concentration of water and land policy in the hands of centralising bureaucracies as necessary for ecological and developmental survival (Alatout, 2008). In South Asia, the ‘Green Revolution’ narrative ostensibly represented a technocratic solution (improved seeds) to long‐standing environmental (loss of soil fertility and yield declines) and economic (food insecurity) problems, but simultaneously legitimised fiscal penalties for ‘backward’ farmers who rejected the high‐input varieties because they feared indebtedness or ecological ruin (Yapa, 1993).

We draw on the fecund lexicon of the multidisciplinary scholarship studying environmental narratives to analyse the cognitional and historical legacies that colour how different actors in (South) Sudan—rebel leaders, commodity traders, Ministry of Agriculture officials, and presidents—‘see’ the interconnectedness between water, energy, and food security. The reproduction of extreme vulnerability to mass hunger cannot be understood without a longitudinal lens that recognises the importance of narratives that expound the ability of Sudanese resources to ‘feed the world’ and supply global markets, but only when organised by foreign and domestic elites in specific ways. Emphasising such continuities and the transmission of ideological constructs around food production and political order across regimes and historical decades does not render political leaders and their external allies as mere captives of structural legacies. As we show, these are agents acting on—and often astutely rekindling—past imaginaries to increase their political power. We centre the role of such imaginaries and narratives in the making of the Sudanese state, before reconsidering the (not so) new politics of food insecurity and economic diversification in South Sudan.

## 3 WATER, SLAVES, AND AGRICULTURAL EXPORTS: BUILDING SUDAN AS A STATE OF HUNGER

From its establishment in the nineteenth century, the modern Sudanese state has been built around a particular imaginary: that control over water meant power and that the ability to grow crops abundantly for export via the Nile River would generate the resources to extend state control over people, land, and territory (Verhoeven, 2015b). This idea of a nexus between water and export agriculture entered Sudan by way of conquest. Invading in 1821, Egypt's ruler Muhammad Ali Pasha wanted to push as far towards the sources of the river as possible given how pivotal it was in his vision of building a European‐style polity. To pay for the infrastructure, a growing bureaucracy, and an army that could fight across the Eastern Mediterranean, Red Sea, and Northeast Africa, Ali and his dynastic successors relied on an export‐oriented economy that sourced especially cotton for European markets and that depended on low‐cost (often corvée) labour and ample water for irrigation (Owen, 1999). Slaves from Sudan—who served in the armed forces, worked on agricultural estates, and acted as legal tender for tax payments (Baer, 1967)—and irrigated production of cash crops lubricated the Egyptian Empire's integration into an industrialising global political economy (Saleh, 2023).

The dependence on land, slaves, and the Nile of the emergent ‘Turkiyya’ colonial state was a function of the vision for political consolidation of the Alawiyya dynasty in Cairo. The reverie also incentivised the imperialists‐cum‐state‐builders to push ever further south. From Khartoum—where the Blue Nile and White Nile merge and therefore the natural capital of a state built around water—the advance towards Equatorial Africa was arduous, but Turkiyya became the first empire to overcome the formidable geographical barrier posed by the Sudd wetlands to claim sovereignty over these hinterlands (Schouten and Bachmann, 2024). Notwithstanding the difficulty of establishing a monopoly on violence in Equatoria, Bahr al‐Ghazal, and Upper Nile, these distant territories became crucial labour reserves in which the presence of state agents was usually coterminous with *zaribas* from whence slave raids, tax collection, and a modicum of local administration were organised from the 1840s and 1850s onwards (Johnson, 2016). Southern Sudan's status as the frontier of the Alawiyya empire disincentivised the state from developing major hydro‐agricultural projects there, but the centrality of slave labour to the Nile‐centric political economy of Egypt and Turkiyya Sudan made the region an integral part of the extractive riverain edifice.

The Turkiyya became associated with large‐scale hunger as agricultural produce was declared a state monopoly and the displacements and violence that Egyptian overrule engendered led to starvation as it disrupted subsistence production and animal herding, especially in Central and Southern Sudan. State‐building also exacted a steep toll in what was fast becoming Sudan's riverain core: famines laid waste to the areas that were compelled to produce irrigated commodities and cattle around Dongola in the 1830s and to Shendi and its cotton and legume fields in the 1860s (Bjørkelo, 2003). Moreover, if punitive taxes and extortionate terms of exchange led Sudanese producers to seek to escape exploitation, hunger also drove the starving to look for food and work, often in those same zones of production where Dinka, Nuer, Shilluk, and slaves from other ethno‐linguistic communities had already been taken to toil. When the 1888–92 ‘Great Famine’ ravaged Sudan following the collapse of Egyptian control, the White Nile and Bahr al‐Ghazal were recurrently raided for food and human beings as northern riverain areas (where Turkiyya dominance had left deep economic scars) sought to convalesce by bolstering their agricultural labour force and slave armies (Pankhurst and Johnson, 1989).

The Anglo‐Egyptian Condominium (1898–1956) and Sudanese governments in the immediate post‐colonial period outlawed slave raiding and encouraged the private sector to play a more prominent role in agricultural production. But the objectives pursued by the state were essentially unaltered: export agriculture remained an indispensable source of revenue and government provision of security and public services was concentrated in riverain Sudan. Periodic food crises continued to underpin the power of elites as the hungry were compelled to sell their assets and work as disposable labour, frequently on lands and schemes owned by those who had garnered fortunes via tax collection and the merciless pacification of the South (Serels, 2013).

In the 1920s, British officials had opened the world's largest centrally‐managed irrigated project, the Gezira Scheme, between the Blue Nile and White Nile to ensure the competitiveness of textiles at home (Barnett, 1977). To grow the vast quantities of cheap cotton needed in Lancashire, free water and land were complemented by seasonal labour from Upper Nile, Darfur, and West Africa (O'Brien, 1983). The resultant rock‐bottom input costs were facilitated by the immiseration of Western and Southern Sudan where barely any public goods were provided and hunger remained rife: this compelled groups that were historically victims of slave raiding to travel to (or settle in) Gezira to eke out a living in the cash economy. One of the only ambitious development projects pioneered in Sudan's Southern districts was the post‐1945 Zande Scheme, which, under strong state guardianship, was heralded as an endeavour in social mobility and food self‐sufficiency. Its operational logic, like in Gezira, reflected the extraverted nostrums of Sudan's political economy: administrators compelled producers to grow cotton for monetary compensation far below international prices. But what made sense for Sudan's state‐builders did not for most of its inhabitants and their food security. While food shortages had not been a problem in Zande District in the past, the resettlement of tens of thousands of Azande disrupted local production. Azande men widely regarded the introduction of cotton cultivation as an instrument to force them to pay taxes to an alien state (Reining, 1966; Onwubuemeli, 1974).

Resistance to profoundly uneven development and the unrepresentative nature of the state intensified. Sudan gained independence amidst the 1955 Torit mutiny that nationalists hail as kickstarting South Sudan's six decades of liberation struggle (Rolandsen, 2011). The escalating violence, especially from 1963 onward, impeded the delivery of public services and complicated possible debate around a reimagined social contract and rekindled understandings of the nexus between food, water, and governance in ways that could benefit local communities. That said, there is little evidence that the latter was in the offing: Sudan's postcolonial elites were politically, financially, and epistemologically wedded to a developmentalism that ignored the crops and animals that the state struggled to instrumentalise for capital formation. They cast Southern deprivation and hunger in racial and religious terms, thereby torpedoing the prospects of consensually forging a nation (Young, 2018).

The coup led by Ja'afar Nimeiri and his leftist army officers generated hope, since their ‘May regime’ wanted to build peace with Southern rebels through the 1972 Addis Ababa Agreement (ending Sudan's First Civil War) and denounced the unimaginative conservatism of Sudan's sectarian forces in economic matters. Yet, their critique was not so much one that questioned the hegemonic imaginary of development as one that decried its weak implementation. The new government doubled down on environmental narratives pertaining to the ‘necessity’ to green Sudan's drylands, the mismanagement of ‘virtually unoccupied’ fertile zones, and ‘lazy’ and ‘ignorant’ smallholders who stood in the way of the Khartoum cognoscenti. Nimeiri dreamed of Sudan being the breadbasket of Africa and the Middle East, an agricultural superpower that would feed its citizens and nurture industries by selling commodities to regional and global markets (Oesterdiekhoff and Wohlmuth, 1983). As Sudan's head of state between 1969 and 1985, Nimeiri nationalised vast lands and reallocated them to those who promised to realise the breadbasket through mechanised agriculture funded with Gulf Arab loans and grants. He opened the Rahad Irrigation Scheme by the Blue Nile and decreed the construction of the pharaonic Jonglei Canal, which would divert Nile water from the Sudd wetlands for irrigation and power‐generating purposes in Egypt and (Northern) riverain Sudan (Collins, 1990).

Sudan, however, did not become the breadbasket of Africa and the Middle East. Nor was it able to develop higher value economic activities while bolstering its own food security. To the contrary, the 1980s and 1990s witnessed massive famines in which hundreds of thousands of people perished—in Darfur (1984–85 and 1990–91), Bahr al‐Ghazal (1985–88, 1990, and 1998), Kordofan (1990–93), Upper Nile (1993), and Red Sea (1990–91) (de Waal, 1997; Keen, 2008). Although droughts were precipitating factors that put huge stress on pastoralist and cultivator communities, desertification, land degradation, and other Malthusian tragedies of the common were not the causes of undernourishment, despite the Sudanese authorities—and many relief organisations—emphasising these environmental narratives (Olsson, 1993; Suliman, 1997). Rather, it was the deadly interplay between conflict, displacement, and looting that drove processes of starvation (Mawson, 1991; Patel, 1994; Deng, 2002). In the context of Sudan's Second Civil War (1983–2005), deadly hunger followed livestock raiding and scorched earth campaigns by the Sudan Armed Forces (SAF) and allied militias in areas that traditionally produced staple foods for local consumption. This deprived communities of their means of subsistence and savings and forced them to seek relief as underpaid agricultural labourers; their assets were transferred to military officers, politically‐connected traders, absentee landlords, and the victims' neighbours enlisted in Khartoum's war efforts—‘the benefits of famine’, in Keen's (2008) powerful phrase.

These mechanics of starvation were underpinned by the growing vulnerability of rural Sudan to such brutal shocks, not by absolute scarcity of output or a lack of emphasis in policies and narratives on food supply—quite the contrary (O'Brien, 1985). Nimeiri's breadbasket fantasies and the self‐sufficiency drive in wheat imposed by the military–Islamist regime that ruled between 1989 and 2019 both reprised the imaginary at the heart of the Sudanese state: top‐down prioritisations of ‘strategic crops’, regressive reallocations of lands, and the expansion of capital‐intensive farming would allow the country to achieve food security while exporting to global markets so that the resultant surplus could strengthen the economy and the groups favoured to dominate it. This obsession with supply meant that both regimes—despite their divergent ideological proclivities—privileged irrigated and mechanised production while neglecting Sudan's ‘traditional sector’ in which three‐quarters of the labour force worked (Verhoeven, 2015b).

It was also during the reign of Nimeiri's government and the military–Islamist regime that energy, principally oil and hydropower, became an integral third leg of the water–food nexus that historically has been crucial to the building of the Sudanese state through processes of violent accumulation and starvation. Oil was discovered in the 1970s, but most confirmed reserves were situated around the colonial North–South border, in areas also targeted in the breadbasket reforms. Nimeiri redrew administrative boundaries to ensure much of the oil was placed in the new ‘Unity’ Province—and therefore under Khartoum's control, rather than that of the post‐1972 autonomous South (Johnson, 2012). During Sudan's Second Civil War, government troops fought in Unity and elsewhere alongside militia predominantly recruited from pastoralist groups that had reaped ‘the benefits of famine’, including in the areas where Chinese investors and labourers would engineer the Greater Nile Oil Pipeline (Sidahmed, 2013, pp. 103–120). Constructed amidst ethnic cleansing in the 1990s (Rone, 2003), the pipeline to Port Sudan on the Red Sea and global markets and other infrastructure established on lands essential to the livelihoods of now starving communities ensured a financial windfall for Khartoum. In the 2000s, billions of petrodollars were reinvested in a dam programme that aimed to transform (urban) Sudan's energy consumption and to expand massively irrigated agricultural output (for exports to the Middle East) as the military–Islamist regime executed its promises to enable core constituencies to prosper. Once more, environmental narratives regarding the ‘idle potential’ of the Sudanese Nile and the profligate stewardship of water and land by rural smallholders were invoked to justify dam building, the expropriation of lands vital for staple food cultivation, and the displacement of tens of thousands in the Northern and Eastern peripheries. Oil and hydropower thus consolidated the traditional model of Sudanese state‐building as reliant on the extraction of resources—not just water and food but now also energy—from the periphery to buttress the core and its external financiers (Terrefe and Verhoeven, 2024).

This was lucrative for a well‐positioned minority, but ruinous for Sudan as a whole, which has since been chronically dependent on food imports and emergency aid to limit the ravages of famine and long‐term malnutrition. On their own terms, the elites' ideas and policies failed, time and again, to deliver the promised development. Yet, the flipside of such food security disasters remained the political utility of these policies: extracting (inter)national resources and structuring rents in this way allowed authoritarian regimes in a historically weak state to deliver material benefits to preferred constituencies, build money‐spinning ties with partners in the West, China, and the Gulf, and consolidate authority at the centre of the polity (Verhoeven, 2015b). Both the military–Islamist and Nimeiri regimes would ultimately collapse amidst mass demonstrations triggered by rising food prices. But they were also, not coincidentally, the two governments with, by far, the greatest political longevity in Sudanese history.

## 4 ‘WE WANT DAMS TOO’: THE (NOT SO) NEW NATIONAL ECONOMY IN SOUTH SUDAN

In September 2023, South Sudan organised its first‐ever National Economic Conference (NEC) in Juba, its capital city. Ever since the 2005 Comprehensive Peace Agreement (CPA) ended Sudan's Second Civil War, the former SPLA/M rebels have governed over the territory, first as an autonomous region and from 2011 as an independent state, relying almost exclusively on oil rents for government expenditure. The NEC stresses economic diversification amidst sustained declines in oil output, emphasising food production and the obligation to ‘[r]evive the national agricultural projects or schemes’ (Republic of South Sudan, 2023, p. 5). Such calls to action continued the key themes of the 2021–24 National Development Strategy, which underlined ‘diversification from petroleum to agriculture’ as the top priority, drawing on WEF nexus‐inspired discourse to connect increased food output to the imperative of ‘[i]nvesting in water as the blue gold economy is paramount for economic recovery and economic diversification’ (Republic of South Sudan, 2021, pp. 48–49). In September 2023, President Salva Kiir invited Russia to establish five hydroelectric dams, including the long‐standing Fula Rapids Hydropower Project close to the Ugandan border, with a price tag of circa USD 1.5 billion and the potential to generate 2,000 megawatts of power (Daniel, 2023).

The NEC and the National Development Strategy reasserted what has since the days of Nimeiri been the standard refrain of South Sudan: a land of possibility—one that boasts substantial assets to deliver on these hydro‐agricultural promises, because 95 per cent of its territory is considered as suitable for agriculture, encompassing 50 per cent of what is classified as prime agricultural land (Japan International Cooperation Agency, 2015). Furthermore, the country experiences abundant rainfall (particularly in Equatoria) and is home to the second‐largest stretch of the Nile River, with many of its smaller tributaries located within South Sudan's borders. The NEC therefore declared that the moment has arrived to ‘switch from relief to development’ now that the 2013–18 conflict, which killed hundreds of thousands of people, is ‘in the past’ and that the SPLA/M can ‘finally’ implement its plans for the nation's abundant yet untapped water, energy, and food resources.^3^ As Kiir tellingly underscored during the inauguration of the Juba water supply system, ‘we are dying of thirst while we are sitting on the Nile . . . although it is just running day and night’ (Eye Radio, 2023).

As a liberation movement allied to leftist comrades in Eritrea, Ethiopia, and Uganda, the SPLA/M was organised in the 1980s and 1990s along the lines of Marxist–Leninist precepts. Its 1983 manifesto committed the movement to a ‘united, democratic and secular Sudan’, seeking a transformation of the existing state rather than secession. It recruited those incensed by racial, religious, and ethno‐linguistic bigotry, but also flew the banner of resistance against exploitative economic policies, especially those pertaining to land, food, and water, by successive governments. The SPLA/M was able to expand its insurgency from Southern Sudan into Kordofan and Blue Nile by gathering support from communities displaced by the expansion of mechanised farming, spurred on by Nimeiri's breadbasket push (Komey, 2008). Moreover, its rebellion abruptly arrested the construction of the Jonglei Canal—the very topic of the PhD thesis of SPLA/M Chairman John Garang and a recruitment booster as the project symbolised to many the discrimination experienced by Southern Sudanese through water and food policies that serve(d) the interests of domestic and foreign elites. Egyptian and Sudanese advocates of the Jonglei Canal disseminated environmental narratives that portrayed the Sudd wetlands as a gargantuan trap of ‘lost’ (evaporating) water which ensnared people living there in disease‐infested, Stone Age‐like conditions. These narratives positioned the waterway as Southerners' chance to enter modern civilisation and dismissed the cultural meanings, ecological functions, and economic benefits the wetlands offer to the Nile Basin and resident communities (el Moghraby and el Sammani, 1985). One of Nimeiri's key allies defended the project in parliament in the following terms: ‘We are not to remain a sort of a human zoo for anthropologists, tourists, environmentalists and adventurers from developed countries of Europe to study us, our origins, our plights, the size of our skulls and the shape and length of customary scars on our forehead’ (Alier, 1974a). Revealingly, he emphasised, regarding the ‘model’ Nimeiri had in mind, that: ‘It is the intention of the Revolution to make this area another Gezira’ (Alier, 1974b). While such grandiloquence resonated in Cairo and Khartoum, it outraged students, pastoralists, and fishmongers (Wawa and Nyibong, 2022), for many of whom, ‘the Sudd is a buffer not only in a geographical sense but also in the sense of a field of force between the centripetal powers of the state and the centrifugal propensities of commitments to mobility and autonomy’ (Schouten and Bachmann, 2024, p. 1041).

The indignation over Jonglei, the breadbasket push, and the centralisation of rents from oil contributed to the SPLA/M's broad appeal across Sudan's peripheries and among the marginalised in the capital, where many hoped that, through the 2005 peace deal, the movement might begin to change the Sudanese economy from within. The mobilisation potential associated with resistance against the environmental narratives propagated by Khartoum elites and their enabling of deleterious policies continued to be recognised well after the CPA, as the updated Manifesto of the Sudan People's Liberation Movement makes clear:

*Uneven and unequal development hast resulted in the irrational use of the country's vast resources and various forms of marginalization (both on ethnic and class bases), including the marginalization of women, impoverishment, deprivation and unequal distribution of wealth and the fruits of growth to regions and peoples of Sudan. This in addition to a skewed pattern of access to and distribution of the basic social services to the disadvantage of the poor and marginalized, both in rural areas and the major urban centers. . . . Serious environmental implications have resulted from the pursuit of this development paradigm and have rendered that development unsustainable. The wanton use of natural resources poses a serious threat to the natural environment, especially in rural areas, in a manner that may lead to the total collapse of the rural economy. Besides, the degeneration of the environment is behind conflicts on marginal grazing lands all over Sudan . . . poverty reduction, and its ultimate eradication, will be the overarching objective of development . . . In order to end urban bias and center‐focused development orientation, the SPLM's vision is to ‘take towns to people in the countryside rather than people to towns’* (SPLM, 2008, pp. 7–8, 21).

During the 2010 elections, before South Sudan's secession, the SPLA/M campaigned against the unequal division of benefits and costs of the military–Islamist regime's dam programme—its flagship economic policy—and nominated the leader of the local resistance against the record‐breaking Merowe Dam, Ali Askouri, as its candidate for the governorship of River Nile State.

However, despite a multitude of historical reasons for the SPLA/M to prioritise a different approach to development, and despite a 2007 policy document insisting that Khartoum's top‐down approach had ‘largely proved ineffective and unsustainable’ (Ministry of Water Resources and Irrigation, Government of Southern Sudan, 2007), once in government there was little evidence of the movement striving to break with the past. To the contrary, its post‐2005 leadership embraced an understanding of development of which clearly its predecessors knew the cost. Indeed, the usefulness of environmental narratives as tools for exercising state power, managing elite politics, and legitimating economic policies would persuade the SPLA/M to implement its version of Sudan's breadbasket strategy.

Following the CPA and the death of its helmsman John Garang in 2005, the liberation movement grappled with insecurities: acrid rivalries between its protagonists (many of whom, including Garang's successor as President, Salva Kiir, and the SPLA/M's new Vice‐President, Riek Machar Teny Dhurgon, waged war against each other during the 1990s), worries about the tenuous power‐sharing arrangement with the SAF and Sudan's Islamists, and uncertainty over how the population would respond to its decisions, all loomed large (Johnson, 2014; Nyaba, 2019). Amidst these anxieties, water, energy, and food policy was rendered subservient to the need to stabilise intra‐elite relations and show select domestic constituencies and foreign partners (whom the SPLA/M wooed to secure its sovereignty) quick results in the building up of an embryonic state. This was apparent from its governance track record prior to independence. The CPA enabled the former rebels to govern Southern Sudan, including broad autonomy in the spending of petrodollars. Yet, the SPLA/M's choices did not fundamentally rupture the long‐standing approach of regimes in Khartoum to food (in)security, water resources, and energy. Instead, they replicated, *mutatis mutandis*, the patterns of elite accommodation and extraversion that have produced hunger and violence for so long—while proving fruitful for the management of internal coalitions and rivals and external partners.

This was evident in the aggressive courting of investors in capital‐intensive agriculture by once more deploying recognisable environmental narratives. Like the military–Islamist‐dominated government, the SPLA/M capitalised on skyrocketing global prices (of food and energy but also of land) in the 2000s and on the interest of Gulf Arab, Asian, and Western investors in ‘finally’ creating a breadbasket. Unsurprisingly, the regional states of Central Equatoria, Jonglei, Unity, and Upper Nile, which surround the White Nile, were central to this ‘new’ geography of hydro‐agricultural development. Hundreds of thousands of hectares were leased to investors who vowed to bring modern technology and cultivation techniques to make the most of the hitherto untapped natural bounties of the land (Hirblinger, 2015). Not only did this routinely involve liberation movement heavyweights partnering with self‐declared ‘cowboy capitalists’ (Pelton, 2014), but these deals also represented an extension of intra‐SPLM competition over influence, money, and ethnic fiefdoms.

In one notorious case, a former Wall Street commodities trader established a joint venture with the son of the SPLA/M's Deputy Commander‐in‐Chief, Paulino Matip, that gave his company, Jarch Capital, a lease over 800,000 hectares in Mayom (close to the oil fields in Unity) to cultivate cereals, oil seeds, and vegetables (Deng, 2011). Also invited on to Jarch's Advisory Board were Vice‐President Riek Machar and generals Peter Gadet and Gabriel Gatwech Chan ‘Tanginye’—all protagonists of ruthless wars over these oil fields and fertile lands. One bitter rival was British energy firm White Nile Limited which contested the rights of both Jarch Capital and France's Total over Sudan's oil Block Ba and entered the agricultural bonanza prior to Jarch under its rebrand, Agriterra (whose founders were accused by Global Witness (2016) of being serial bribers and frauds). Before calling itself Agriterra, White Nile Limited was 50 per cent owned by Nilepet, the SPLA/M's institutional vehicle to make deals with oil companies during the liberation war and later the national and gas company of South Sudan; Edward Lino (one of the SPLA/M's founding members) and Lual Deng (the SPLA/M's chief economic negotiator as well as (united) Sudan's Minister of Oil) served on White Nile Limited's Board (*Sudan Tribune*, 2008). Notwithstanding the ways in which such extractive schemes threatened local access to food, the speculative fever over land during the CPA years showcased how soaring global commodity prices were once again leveraged in power struggles, this time in a self‐governing Southern/South Sudan. Ironically, but not coincidentally, economic policy in the South reflected the very Sudanese practice of interlacing state authority and private elite interests through water, energy, and food.

From 2005 to the recent NEC, SPLM/A grandees have habitually juxtaposed a caricature of South Sudan's past—a land of hunger, idle resources, and virgin acreage—with the beckoning future of record harvests and economic sovereignty via foreign investment. Reflecting the enduring power of the environmental narrative that built the modern Sudanese state, Salva Kiir repeatedly urged ‘investors worldwide to come and invest in Southern Sudan. In terms of agriculture, it will be the bread basket of the region and the world’ (CMI, 2010). Kiir was by no means the first prominent Southerner to adopt such familiar ‘Sudanese’ discourse about putting water, land, and energy at the disposal of outsiders in exchange for political and financial resources. Abel Alier, a Southern lawyer who served as Vice‐President in Khartoum between 1971 and 1982, passionately defended Nimeiri's hydro‐agricultural ambitions, adopting the rhetoric of his head of state to make the case for the Jonglei Canal. He also echoed the authoritarianism and violence that historically has accompanied this environmental narrative, warning in parliament that ‘[i]f we have to drive our people to paradise with sticks, we will do it’ (cited in Waterbury, 1979, p. 77). The SPLA/M rebelled in the 1980s against the regime that Alier had served, but many of its leaders were familiarised with these hegemonic ideas around development and power during their studies at the University of Khartoum, just like Alier. The Faculty of Engineering was especially influential, including through its education of Lam Akol (later Foreign Minister of Sudan for the SPLA/M) and Riek Machar (Vice‐President of South Sudan, 2011–13, 2016, and 2020–), but the faculties of Economics and Law were too: future SPLA/M ministers (such as Lual Deng, Michael Makuei Lueth, and Nhial Deng Nhial) were immersed in ideas surrounding a nexus between water, agriculture, and power that were broadly shared in Khartoum circles. These beliefs were also inculcated in those who obtained scholarships to attend university in Egypt where similar storylines were (and are) hegemonic. One Southerner, who studied agronomy in Cairo and after 2005 became Director‐General of the SPLA/M‐controlled Ministry of Agriculture and Forestry, still reminisces about writing a dissertation on the theme of ‘Egypt is the Nile; the Nile is Egypt’,^4^ reflecting deeply ingrained ideas regarding state‐building and the environment in the minds of elites.

This cognitive legacy has infused not only the SPLA/M's approach to agriculture, but also its growing interest in dams. Since 2009, when the extremely powerful Dams Implementation Unit in Khartoum convinced Southern officials of the merits of the Fula Rapids Hydropower Project, dams have been integral to a broader prioritisation of large‐scale infrastructure, spanning bridge building over the Nile to plans for massive electrification, the construction of the Juba River Port, a pipeline to Djibouti, and the rehabilitation of the Wau–Aweil–Port Sudan railroad. The construction of dams is perceived as a validating stamp for South Sudan's entry into the league of developed nations. As a senior official at the Ministry of Agriculture and Food Security purported: ‘Uganda, Ethiopia, Egypt or Sudan have dams; it is our turn now’.^5^ Constructing dams would also assert South Sudan's hard‐fought sovereignty, allowing it to ‘activate its fundamental right to utilise the Nile’,^6^ thereby establishing South Sudan as a significant player in regional geopolitics. Dam building, whether economically appropriate or not, is hence a means of leveraging South Sudan's share of Nile waters for enhancing its sovereignty (Verhoeven and Sennesael, 2022). Civil servants recall meetings in which South Sudan feels consistently looked down upon by its neighbours, viewed as ‘young and incapacitated’. ‘It is time’, one senior civil servant insisted, ‘to be taken seriously on the regional stage’.^7^

While it remains to be seen if and when the five dams that were announced in 2023 will materialise, their announcement exemplifies the SPLA/M's eagerness to craft its own nexus between water, energy and food. Dams are asserted to be ‘much more than irrigation’.^8^ Most electricity in South Sudan is currently produced using diesel generators. Although the government is negotiating agreements with Ethiopia and Uganda to import power, the SPLA/M emphasises the need for South Sudan to become a producer of electricity and for dams to become symbols of (aspirational) self‐sufficiency in food and energy in a region marked by a history of rapidly shifting political alliances. Senior figures in the SPLA/M believe the party cannot rest on its laurels and needs to use water, energy, and food politically because, in the words of one former Minister of Oil, ‘your friend can become your enemy overnight’.^9^

Aspirations for birthing a ‘new’ domestic political economy are therefore intimately tied to the desire to link South Sudan to regional and international circuits in ways that can enhance the government's influence. The SPLA/M has fervently championed South Sudan's agricultural potential with its neighbours, drawing on well‐known environmental narratives to point out how it can help to tackle their internal crises. For instance, in May 2023, Minister of Agriculture and Food Security Josephine Lagu purported that South Sudan had ‘the capacity to produce food in [excess] to sell to the neighbouring countries and beyond’ (Johnson, 2023). In its biannual dialogue with Cairo, Juba has highlighted the threats posed by climate change to Egyptian‐irrigated agriculture, contrasting it with South Sudan's ‘unique’ potential and abundant rainfall. Juba has also professed its ability with regard to feeding Sudan's war‐torn peripheries and absolving Khartoum of that historical ‘burden’. Meanwhile, in engagements with Addis Ababa, the SPLA/M has reiterated its ‘duty’ to help Ethiopia address its rampant demographic growth and high inflation by offering to give it priority access to Southern Sudanese food imports—an essential service that Juba stands ready to provide.^10^ Positioning South Sudan as essential to the regional political economy of water, energy, and food, also dovetails with global angst about commodity shortages and price hikes driven by geopolitical polarisation and climate change. Especially since the 2022 outbreak of the Russia–Ukraine conflict and soaring energy and food prices worldwide, the SPLA/M government has remarketed the attractiveness of its oil industry and is trying to seduce outside capital by once again publicising its ‘ample’ water resources and ‘unparalleled’ potential in agricultural production.

The gap between such external posturing and the situation on the ground remains shocking, even for seasoned observers of the cut‐throat politics of the Horn of Africa. In the aftermath of the CPA, the SPLA/M's official policy documents underscored ‘the significance of changing approaches to water sector development during the 1980s and 1990s and [the need] to take account of lessons learned’ to increase food security (Government of Southern Sudan, 2007, p. 6). However, apart from a few token projects funded by international donors to empower local farmers, no efforts have been undertaken to effectively lay the groundwork to decrease vulnerability to external shocks. The breadbasket discourse notwithstanding, agricultural productivity remains very low and subsistence producers and pastoralists continue to receive almost no assistance to increase local yields or to organise themselves. While the Comprehensive Agricultural Development Master Plan, finalised in 2017, once more emphasised the necessity of supporting smallholders, its implementation has remained virtually nonexistent.

Indeed, six years after the end of the civil war, and despite frequent promises of a green revolution in South Sudan, the food security situation remains catastrophic. In 2023, the country received more than USD 1.1 billion in humanitarian aid. The share of the population having access to drinkable water has stagnated at around 41 per cent since independence and puddles and rivers remain the primary source of water consumption for most citizens (World Bank Group, 2024); in 2021–22, South Sudan battled its worst floods on record, rendering cultivation impossible in Unity and Upper Nile Provinces. The latest IPC analysis projects that about two‐thirds of the population will experience severe food insecurity between April and July 2024, an increase of one million as compared to 2018. While the cereal deficit has decreased since 2018, it is estimated that in the January–December 2023 marketing year, the deficit still stands at a whopping 485,400 tonnes, which translates into a massive (and rising) food import bill (FAO, 2024). In March 2023, the International Monetary Fund bailed out the government with emergency financing through the Food Shock Window of the Rapid Credit Facility. By August 2023, South Sudan was facing ‘the highest levels of hunger in the world’ (United Nations, 2023). It is hard to argue that this is in spite of the imaginary of South Sudan as a regional breadbasket, rather than because of it.

## 5 CONCLUSION

According to the World Bank (Andree et al., 2024), ‘food security is likely to remain one of the critical challenges for the world to face’. Yet, even though more than 300 million people are suffering acute malnutrition worldwide in early 2024, the prominence of famine prevention on policymakers' agendas has been at best fluctuating, reflecting the abiding assumption that food insecurity is cyclical and primarily the result of hard to anticipate shocks. The tendency to ‘continue to blame it on the rain’, as Sandstrom and Juhola (2017) put it, means that international responses remain predominantly humanitarian and geared towards emergency relief: they continue to be rooted in simplified accounts of complex processes that habitually overlook the often‐long history of recurrent food crises and how vulnerable populations themselves characterise their struggles to access the desired calories. These are profoundly political questions that require the bringing together of the material circulation of resources with cognitive perspectives. In that vein, recent scholarship on Egypt draws attention to the social construction of ‘staple security’ (Barnes, 2022). This happens from below, in that anxieties across social classes about accessing staples have helped spawn a bureaucratic behemoth that is labouring, everywhere and constantly, towards supplying people's daily bread, as government officials fearful of mass instability believe that they need to be seen to respond to both the physical and affective dimensions of hunger. But quotidian fears of staple insecurity are also fed from above, because the speech act of security enables state interventionism in environments, domains of social life, and agricultural processes that goes far beyond its ostensible objectives and reproduces the very anxieties that justified its initial claims to exceptional authority.

This paper has underscored the importance of ideas about the environment, development, and power in making and remaking food insecurity in ‘spaces of vulnerability’. We highlighted the enduring legacy and consequent impacts of hegemonic environmental narratives and how they enable specific policies and broader state‐building processes. Deconstructing these narratives and their material effects compels a reckoning with cognitive legacies, power dynamics, and historically‐grounded processes of knowledge production which, we argue, contribute to the (re)production of food insecurity across regimes and eras.

The (not so) new politics of ‘the’ water‐energy‐food nexus in South Sudan evince our argument. We explored why the SPLA/M, despite decades of pledges to ‘break from the past’ and adopt bottom‐up approaches to tackle food insecurity, is pushing the very policies the party previously opposed, even though this is rekindling a profound vulnerability to mass hunger. To do so, we pursued a critical approach to transcend apolitical and ahistorical perspectives. Understanding how environmental scarcity and/or abundance are interpreted in the South Sudanese context cannot be divorced from the pivotal, enduring role played by imaginaries around water, energy, and food in the building of political orders. Such discourses ‘served, and continue to serve, important functions for particular groups’ (Keen, 1991, p. 150). Contemporary SPLA/M tropes are deeply rooted in nineteenth and twentieth century environmental narratives regarding the Nile and agriculture and their potential for power consolidation. The crux of the issue is therefore not the often‐woeful management of ambitious schemes; rather, it lies in the perpetuation of hegemonic discourses regarding the problems to be addressed and their proposed solutions—even if these have consistently proven detrimental to the many throughout recent history and primarily benefited a few.

Within the international development literature, the unilateral imposition of Western‐crafted paradigms of neoliberal modernisation on febrile African societies is a frequently recurring theme. However, the last centuries of Sudanese history demonstrate the salience of locally‐crafted hegemonic environmental narratives surrounding the causes of, and proposed solutions to, persistent mass hunger. These have significantly influenced international policies, frameworks, and silences, and continue to do so. National elites' discourses on the WEF nexus and the geography of starvation in Sudan and South Sudan have frequently been co‐opted by donors, embracing the ‘breadbasket’ narrative of the government. This happens not only at the level of rhetoric—for instance, in August 2023, the Director‐General of the FAO, Qu Dongyu, repeated as fact that ‘South Sudan has the potential to be the breadbasket of east Africa’ (United Nations, 2023)—but also in day‐to‐day programming—such as the United States Agency for International Development's post‐2005 efforts to promote commercial farming in South Sudan's ‘greenbelt’ and the Norwegian government's consideration of funding for the Fula Rapids Hydropower Project.

The global significance of the Sudanese case, and more broadly the Horn of Africa, in rethinking starvation and its prevention should not be underestimated. Our analysis can serve as a reminder of the importance of paying heed to the ideas that, explicitly or implicitly, structure our approach to water, energy, and food and shape the realm of the possible and the (un)desirable, whether in (South) Sudan or elsewhere. Famine prevention continues to be operationalised based on the notion of ‘technical solutions to technical problems’. This approach, which draws strength from deterministic and generalising global discourses on climate change, food scarcity, and underdevelopment, is evident in the proliferation of early warning systems, weather forecasting tools, and conflict prediction mechanisms as the ‘solution’ to food insecurity. However, while technology or strong institutions can, of course, in some cases contribute to mitigating severe hunger, as Ferguson (1994, p. 162) reminds us, ‘the question of power cannot be written off quite so easily’. Understanding how the *problématique* is framed and by whom is crucial for challenging existing policies, institutions, and narratives that are at the root of decades of hunger.

## ETHICS STATEMENT

This paper reports analysis of primary data. Persons from whom data were collected gave their free, prior and informed consent. The data has been kept confidential and used anonymously.

## Data Availability

Data sharing is not applicable to this article as no new data were created or analyzed in this study.

## Endnotes

^1^
Coronavirus disease 2019.

^2^
The Integrated Food Security Phase Classification (IPC) is a standardised tool used globally to assess and classify the severity of food insecurity and malnutrition situations, considering various indicators including food consumption, livelihood changes, nutritional status, and mortality rates. The classification ranges from Phase 1 (minimal), in which households can meet essential food and non‐food needs, to Phase 5 (catastrophe/famine), meaning that households experience an extreme lack of food and/or other basic needs even after the full employment of coping strategies.

^3^
Interview with a Minister of the Transitional Government, October 2023.

^4^
Interview with an anonymous source, October 2023.

^5^
Interview with a civil servant from the Ministry of Agriculture and Food Security, Juba, October 2023.

^6^
Interview with a civil servant from the Ministry of Agriculture and Food Security, Juba, October 2023.

^7^
Interview with civil servants from the Ministry of Agriculture and Food Security, Juba, October 2023.

^8^
Interview with a civil servant from the Ministry of Agriculture and Food Security, Juba, October 2023.

^9^
Interview with a former State Minister for Finance, Washington, DC, October 2020.

^10^
Interview with a civil servant from the Ministry of Water and Irrigation, Juba, October 2023.
